# Quantitative analysis of proteome extracted from barley crowns grown under different drought conditions

**DOI:** 10.3389/fpls.2015.00479

**Published:** 2015-06-30

**Authors:** Pavel Vítámvás, Milan O. Urban, Zbynek Škodáček, Klára Kosová, Iva Pitelková, Jan Vítámvás, Jenny Renaut, Ilja T. Prášil

**Affiliations:** ^1^Division of Crop Genetics and Breeding, Plant Stress Biology and Biotechnology, Crop Research InstitutePrague, Czech Republic; ^2^Faculty of Forestry and Wood Sciences, Czech University of Life Sciences PraguePrague, Czech Republic; ^3^Department of Environmental Research and Innovation, Luxembourg Institute of Science and TechnologyBelvaux, Luxembourg

**Keywords:** *Hordeum vulgare*, crown, drought, proteomics, phenotyping candidate

## Abstract

Barley cultivar Amulet was used to study the quantitative proteome changes through different drought conditions utilizing two-dimensional difference gel electrophoresis (2D-DIGE). Plants were cultivated for 10 days under different drought conditions. To obtain control and differentially drought-treated plants, the soil water content was kept at 65, 35, and 30% of soil water capacity (SWC), respectively. Osmotic potential, water saturation deficit, ^13^C discrimination, and dehydrin accumulation were monitored during sampling of the crowns for proteome analysis. Analysis of the 2D-DIGE gels revealed 105 differentially abundant spots; most were differentially abundant between the controls and drought-treated plants, and 25 spots displayed changes between both drought conditions. Seventy-six protein spots were successfully identified by tandem mass spectrometry. The most frequent functional categories of the identified proteins can be put into the groups of: stress-associated proteins, amino acid metabolism, carbohydrate metabolism, as well as DNA and RNA regulation and processing. Their possible role in the response of barley to drought stress is discussed. Our study has shown that under drought conditions barley cv. Amulet decreased its growth and developmental rates, displayed a shift from aerobic to anaerobic metabolism, and exhibited increased levels of several protective proteins. Comparison of the two drought treatments revealed plant acclimation to milder drought (35% SWC); but plant damage under more severe drought treatment (30% SWC). The results obtained revealed that cv. Amulet is sensitive to drought stress. Additionally, four spots revealing a continuous and significant increase with decreasing SWC (UDP-glucose 6-dehydrogenase, glutathione peroxidase, and two non-identified) could be good candidates for testing of their protein phenotyping capacity together with proteins that were significantly distinguished in both drought treatments.

## Introduction

Drought, which significantly reduces agricultural production, represents the most severe abiotic stress worldwide. There are several definitions of drought based on different views and constraints such as meteorological drought, physiological drought, etc. (Lawlor, 2013). Physiological drought represents a discrepancy between plant water uptake and water release, resulting in water deficit and cellular dehydration. Cellular dehydration induces profound alterations in plant cell structure and metabolism, aimed at minimizing the harmful effects of the drought. Drought induces several processes in plant cells including: increased levels of abscisic acid, the levels of some metabolites such as proline, induction of stress-regulated genes, and changes in the activity of some proteins (Kosová et al., 2011). Proteins play an important role in plant adjustment to water deficit since they are directly involved in plant cell structure and metabolism. Stress-induced proteins include regulatory proteins (e.g., transcription factors, protein kinases, protein phosphatases, signaling proteins), as well as effector proteins directly involved in stress tolerance acquisition (such as chaperones), late embryogenesis abundant (LEA) proteins (such as dehydrins), mRNA-binding proteins, water channel proteins, osmolyte synthesis enzymes, components of protein biosynthesis and degradation, cytoskeletal proteins, and detoxification enzymes (Kosová et al., 2011). Dehydrins belong to the LEA protein family, and are induced by low-temperature, drought, and salinity stress in plants (Kosová et al., 2014). Moreover, in our previous studies, the accumulation of dehydrins was used to correlate cereal genotypes with different tolerance levels to abiotic stresses (Vítámvás et al., 2007, 2010; Ganeshan et al., 2008; Kosová et al., 2008, 2010, 2012, 2013; Vítámvás and Prášil, 2008; Holková et al., 2009). However, the resulting level of abiotic stress tolerance also depends on components of plant stress response other than dehydrin accumulation; therefore, detailed knowledge about the stress-dependent proteome changes is necessary.

Plant response to drought can be very diverse, depending on the severity of stress and stress timing with respect to the plant's developmental phase. Plant stress response represents a dynamic process where several phases with unique proteome compositions can be distinguished (Levitt, 1980; Larcher, 2003; Kosová et al., 2011). The initial phases of stress response (alarm and acclimation phases) usually reveal more profound differences in proteome composition (with respect to the controls), compared to later phases of stress (tolerance phase) when a novel homeostasis between plant and environment has already been established.

Barley (*Hordeum vulgare*) is a relatively drought- and salt-tolerant cereal crop having originated in semi-arid regions of the Middle East. Recent publication of the complete barley genome sequence (The International Barley Genome Sequencing Consortium, 2012) has significantly improved the accuracy of protein sequence identification, thus enhancing the reliability of the proteomic results. Therefore, barley represents an ideal model for investigation of crop proteome response to several stress factors. Several studies have compared barley's response to drought at the transcript level (Ueda et al., 2004; Talame et al., 2007; Tommasini et al., 2008; Guo et al., 2009). However, the accumulation of transcripts only gives a rough estimation of the protein accumulation due to translational regulations and post-translational modification or degradation of the protein (Kosová et al., 2011). Barley proteome response of barley organs to drought has only been studied by a few researchers (leaf and root—Wendelboe-Nelson and Morris, 2012; leaf—Ashoub et al., 2013; leaf—Ghabooli et al., 2013; shoot—Kausar et al., 2013). Moreover, until now, no study of changes in barley crown proteome under drought was performed. In cereals, it has been shown that survival of a plant depends on the survival of its crown tissues (e.g., Tanino and McKersie, 1985), since crowns contain both root and shoot meristems, and thus are crucial for both root and shoot regeneration after stress treatment. Due to a lack of RuBisCO, many more protein spots could be detected and analyzed by the gel-based proteomic approach than from leaf samples (e.g., Hlaváčková et al., 2013).

The aim of the study was to investigate the response of spring barley Amulet to drought at two different intensities of drought stress, characterized by different levels of soil water capacity (SWC): 35% SWC—mild drought—D1; 30% SWC—severe drought—D2; and 65% SWC—control—C. Therefore, our study had the following partial goals: (1) To investigate barley cv. Amulet response to two differential levels of drought characterized by plant water relationships (water saturation deficit, osmotic potential), the effect of drought on photosynthesis and water use efficiency characterized by ^13^C discrimination, and total proteome analysis by two-dimensional difference gel electrophoresis (2D-DIGE). (2) To compare the effects of two intensities of drought stress on barley plants with respect to the severity of stress impacts on the plant characteristics described above. Detection and identification of barley crown proteins revealing a differential abundance between control and drought, as well as between the two drought treatments associated with a determination of basic plant water characteristics; enabling us to distinguish common processes underlying barley's response to drought as well as specific processes differentiating the two drought intensities.

## Materials and methods

### Plant materials and growth conditions

The experiments were performed on spring barley cv. Amulet (*Hordeum vulgare* L.) obtained from the Gene Bank of the Crop Research Institute in Prague (Czech Republic). Amulet is an important spring malting barley cultivar grown in the Czech Republic (see detailed characterization and pedigree at http://genbank.vurv.cz/genetic/resources/asp2/default_a.htm). The seeds were germinated at 20°C for 3 days in darkness. After germination, the seedlings (9 plants per 8 L pot) were grown in soil (a mixture of Alfisol with manure and sand, 6:2:1) under controlled conditions in a greenhouse (20°C with 16/8 h of light/dark provided by a high-pressure sodium lamp with an irradiation intensity of 450 μmol·m^−2^·s^−1^). The humidity of the soil was maintained at 65% of soil water capacity (SWC), with watering of the pots each day to maintain a constant weight (5500 g). Under these conditions, the plants were grown to the stage of the full development of the 2nd leaf. Next, one third of the plants were kept under this optimal watering (C); while the other plants had water withheld until the SWC reached 35% in the second third of pots, and 30% in the last third (5 and 6 days, respectively) under the same growth conditions. For the next 10 days (9 days for D2) the plants were watering at these SWS levels to reach plants grown at the three levels of SWS: C (65%), D1 (35%), and D2 (30%). Next, the youngest but fully-developed leaf was sampled for water-related parameters and for content of dehydrins; and the crowns for 2D-DIGE analysis. At least three biological and technical replicates of the leaves and crowns were harvested for these analyses. Samples were taken during the fourth hour of the light period. Samples for dehydrin content and 2D-DIGE analysis were immediately frozen in liquid nitrogen and kept at −80°C.

### Water saturation deficit (WSD)

Immediately after sampling the leaves were cut into 1 cm long segments. After measurement of their weight (initial weight; Wi) on an analytical scale, the segments were fully water saturated in polyurethane cells for 3 h and weighed (weight after saturation; Ws). Afterwards, the segments were dried overnight at 95°C and the dry weight (DW) was measured. WSD (%) was calculated as WSD = 100 × (Ws-Wi)/(Ws-DW).

### Osmotic potential (OP)

The leaves were inserted into a sterile syringe and isolated by Parafilm PM-992 (Bemis). Syringes were kept at −80°C in a freezer. Afterwards, the sample was defrosted at room temperature before the measurement of OP. The liquid needed for OP measurement on a HR 33T Dew Point Micrometer (Wescor) was obtained by pressure on the leaves in the syringe.

### Carbon isotope ^13^C discrimination

Leaves for isotope analysis were dried (80°C until of constant weight), ground in a Micro ball mill MM 301 (Retsch). Discrimination of ^13^C was measured by an IsoPrime High Performance Stable Isotope Ratio Mass Spectrometer (GV Instruments), connected with an Euro EA 3200 analyser (Eurovector), according to the manufacturer's instructions.

### Accumulation of dehydrins

Dehydrin accumulation was investigated by immunoblotting of protein soluble upon boiling with anti-dehydrin antibody (Enzo Life Sciences) described by Vítámvás et al. (2010). In short, proteins soluble upon boiling were extracted by Tris buffer [0.1 M Tris-HCl, pH 8.8, containing complete EDTA-free protease inhibitor cocktail (Roche)], from frozen leaves ground in a mortar and pestle under liquid nitrogen. After the boiling step (15 min), the proteins were precipitated under acetone with 1% ß-mercaptoethanol. The protein concentrations were determined utilizing a 2-D Quant kit (GE-Healthcare). About 2.2 μg of the extracted proteins were loaded into each well of 10% SDS-PAGE (Laemmli, 1970). The proteins were electrophoretically transferred to nitrocellulose (0.45 μm; Pharmacia Biotech). The anti-dehydrin antibody, bound to the protein bands, was visualized by BCIP/NBT staining (Bio-Rad). A GS-800 calibrated densitometer (Bio-Rad) was used for image capture of the visualized dehydrin bands. Densitometric quantification of the dehydrin bands was done by Quantity One version 4.6.2 software (Bio-Rad).

### 2D-DIGE analysis

Protein extraction from the frozen crowns was carried out as described by Wang et al. (2006) with some modifications. Briefly, 200 mg of crowns (i.e., 9–12 plants) were ground in liquid nitrogen to a fine powder in 1 mL cold TCA in acetone with 0.07% DTT. After 30 min of incubation in a freezer (−20°C), the homogenate was centrifuged (10,000 × g; 5 min; 4°C); next, the supernatant was decanted and the pellet was washed twice (10,000 × g; 3 min; 4°C). After overnight drying of the pellet at room temperature, the pellet was re-suspended in 0.7 mL phenol (Tris-buffered, pH 8.0) and 0.7 mL SDS buffer (30% sucrose, 2% SDS, 0.1 M Tris-HCl, pH 8.0, 5% 2-mercaptoethanol), and then thoroughly vortexed and centrifuged (10,000 × g; 3 min). The upper phenol phase was added to cold methanol with 0.1 M ammonium acetate (1:5 volume ratio), kept 30 min at −20°C, and then centrifuged (10,000 × g; 5 min; 4°C). The supernatant was discarded. The pellet was washed twice with cold methanol with 0.1 M ammonium acetate, and twice washed with 80% acetone (10,000 × g; 3 min; 4°C). The pellet was dried and dissolved in lysis buffer (30 mM Tris pH 8.0, 7 M urea, 2 M thiourea, 4% w/v CHAPS). The pH of the lysate was adjusted to 8.5 by the careful addition of 50 mM NaOH, and the protein concentration was quantified by use of a 2-D Quant kit (GE Healthcare). Protein extracts were labeled (with dye switching between repetitions) prior to electrophoresis with the CyDyes™ (GE Healthcare) according to the manufacturer's instructions. Ninety micrograms of the proteins (30 μg of each sample plus 30 μg of internal standard) were loaded on each gel and separated by 2-DE (O'Farrell, 1975). Isoelectric focusing was run on ReadyStrip™ IPG strips (pH 4–7, 24 cm; Bio-Rad) on a PROTEAN IEF cell (Bio-Rad) according to the manufacturer's instructions until 90,000 V h were reached. The rehydration buffer contained 9.8 M urea and 4% CHAPS. After equilibration of IPG strips in equilibration buffer with DTT, and then with iodoacetamide, the focused proteins were separated in the second dimension by 12.5% SDS-PAGE (Laemmli, 1970). SDS-PAGE was performed in Ettan DALT six (GE Healthcare). Image capture of the gels was done using the PharosFX Plus (Bio-Rad) at a resolution of 100 μm.

Densitometric analysis of the scanned images was carried out using PDQuest Advanced 8.0.1 (Bio-Rad). Protein spot normalization was carried out using the local regression model, and the spot manual editing was carried out using the group consensus tool. The differentially abundant protein spots (at least a two-fold change; *p* = 0.05) were chosen for spot excision (ExQuest Spot Cutter; Bio-Rad), and identification from preparative gels (2-DE of 750 μg of internal standard sample) were stained by Bio-Safe Coomassie G-250 stain (Bio-Rad).

Protein identification was carried out by MALDI-TOF/TOF. After washing and desalting in ammonium bicarbonate 50 mM/50% methanol v/v, followed by 75% ACN v/v, the spots were then digested *in situ* with Trypsin Gold (mass spectrometry grade, Promega, 10 mg/mL in 20 mM ammonium bicarbonate) using an Ettan Digester robot (GE Healthcare) from the same workstation. Automated spotting of the samples was carried out with the spotter of the Ettan Spot Handling Workstation (GE Healthcare). Peptides dissolved in a 50% ACN containing 0.5% TFA (0.7 mL) were spotted on MALDI-TOF disposable target plates (Applied Biosystems) before the deposit of 0.7 mL of CHCA (10 mg/mL ACN 50% v/v/TFA 0.1% v/v, Sigma Aldrich). Peptide mass determinations were carried out using an Applied Biosystems 5800 Proteomics Analyzer (Applied Biosystems). Both the peptide mass fingerprinting and tandem mass spectrometry (MS/MS) analyses in reflection mode were carried out with the samples. Calibrations were carried out with the peptide mass calibration kit for 4700 (Applied Biosystems). Proteins were identified by searching against the SWISS-PROT, TREMBL, NCBI, and a wheat expressed sequence tag database generated from the databases using MASCOT [Matrix Science, http://www.matrixscience.com; NCBInr downloaded on June 6, 2014 (40,910,947 sequences; 14,639,572,021 residues); EST_monocots downloaded on December 16, 2013 (45,575,892 sequences; 7,829,773,678 residues)]. All searches were carried out using a mass window of 100 ppm and a fragment mass window tolerance of 0.5 Da, and with “Viridiplantae (Green Plants)” as taxonomy for the NCBI database (1,717,798 sequences). The search parameters allowed for the carboxyamidomethylation of cysteine, dioxidation of tryptophan, and oxidation of methionine. Homology identification was retained with the probability set at 95%. All identifications were manually validated. Protein spots containing more than one significantly identified protein were excluded from further analysis. In the case of protein sequences identified as “predicted protein” with an unknown function, protein BLAST search (BLASTP) was carried out against the NCBInr database [NCBInr 20150217 (61,023,628 sequences) and UNIPROT database (UniProtKB Protein) generated for BLAST on Feb 2, 2015 (91,447,086 sequences)] to find an identified protein revealing a significant sequence similarity. Theoretical pI and MW values were calculated from the identified sequence in NCBInr using ExPASy tool (www.expasy.org). The protein functions were assigned using a protein function databases Inter-Pro (http://www.ebi.ac.uk/interpro/), Pfam (http://pfam.janelia.org/) and Gene Ontology (http://www.geneontology.org/).

### Statistical analyses

For each treatment, the statistical analysis was carried out with at least three biological replicates for proteomics, three biological and three technical replicates for WSD, OP, discrimination of ^13^C, and dehydrin accumulation analysis. By analyzing protein abundance values, only statistically significant results were considered (One-Way and Two-Way analysis of variance (ANOVA), *p* < 0.05), and differentially abundant proteins with a ratio of at least 2.0 in absolute value, observed in at least one condition, were selected. A principal component analysis (PCA) was run on the protein spots matched on the different spot maps for qualitative appreciation of the proteomic results. Two-Way ANOVA and PCA were performed on Statistica version 10 software (StatSoft). For detailed analysis of proteome changes, the protein ratio was calculated between treatments and statistically analyzed by Student's *T*-test (*p* < 0.05). All spot densitometric data from samples grown under D1 and D2 were also used as one data set to obtain common plant responses to the drought condition (D). Cluster analysis of the protein spot relative abundance of selected protein spots has been carried out using PermutMatrix software (version 1.9.3; Caraux and Pinloche, 2005). For cluster analysis, Z-score transformation of spot density data was carried out. Euclidean distances and Ward's minimum criteria were used for the analysis.

## Results

### Physiological characterization of barley plants

**Figure 1:**
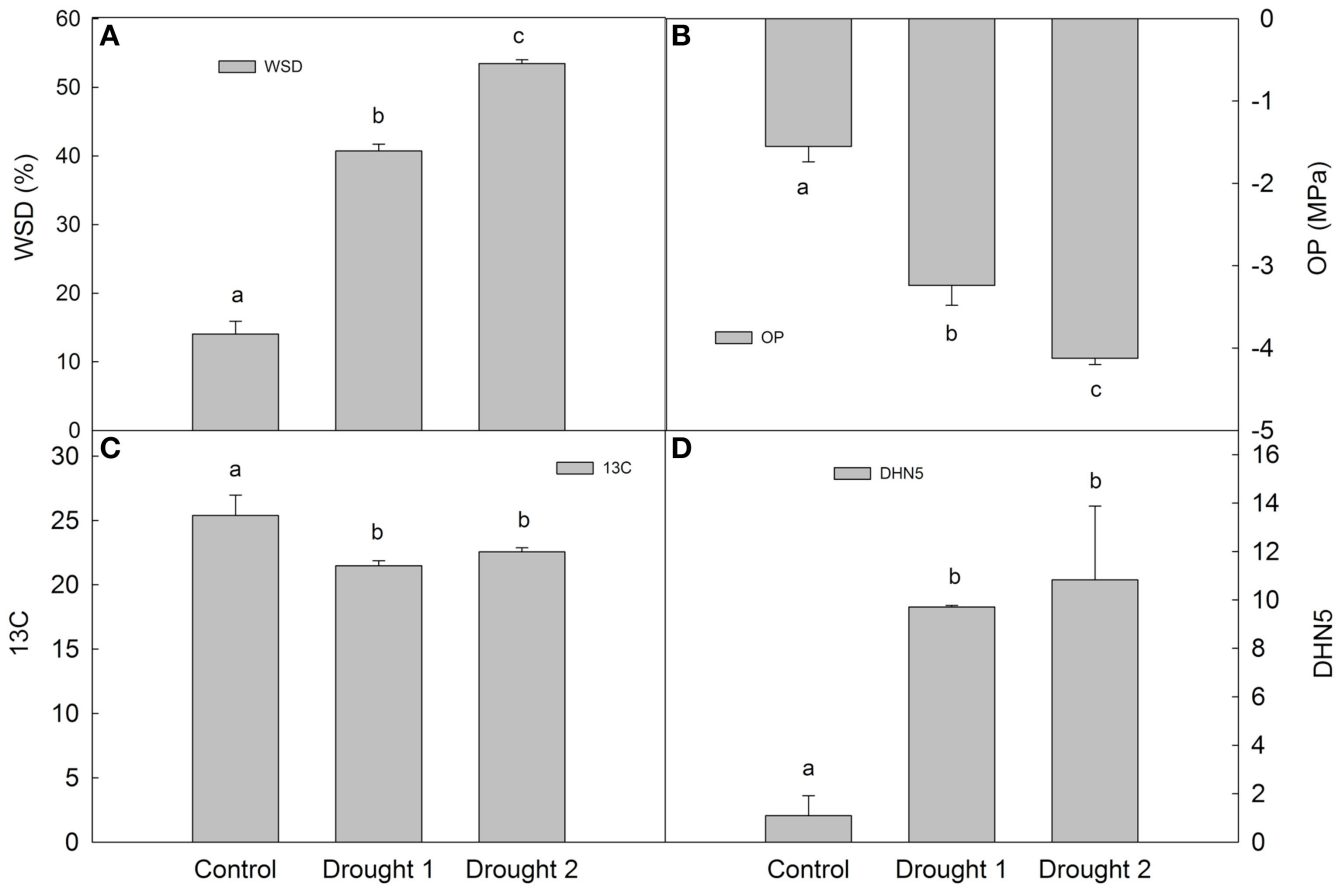
**Water saturation deficit (WSD; A), osmotic potential (OP; B), ^13^C discrimination (13C; C) and dehydrin 5 (DHN5) relative accumulation (D) in Amulet plants sampled under control, drought 1 and drought 2 conditions**. Error bars indicate standard deviation (SD), different letters indicate significant differences at 0.05 level using Duncan's multiple range test.

In barley leaves, the following physiological characteristics were determined at each sampling (in each experimental variant): water saturation deficit (WSD), osmotic potential (OP), ^13^C discrimination (Δ^13^C; Figure 1). Drought led to an increase in WSD and a decrease in OP, with D2 leading to higher dehydration than D1 (milder drought). Regarding dehydrin DHN5 relative accumulation and Δ^13^C, there was a significant increase in DHN5 and a significant decrease in Δ^13^C upon drought with respect to the control; however, there were no significant differences between the two drought treatments. Plants grown under drought conditions revealed slower growth and development than plants under optimal watering (C) conditions.

### Proteomic analysis

**Figure 2:**
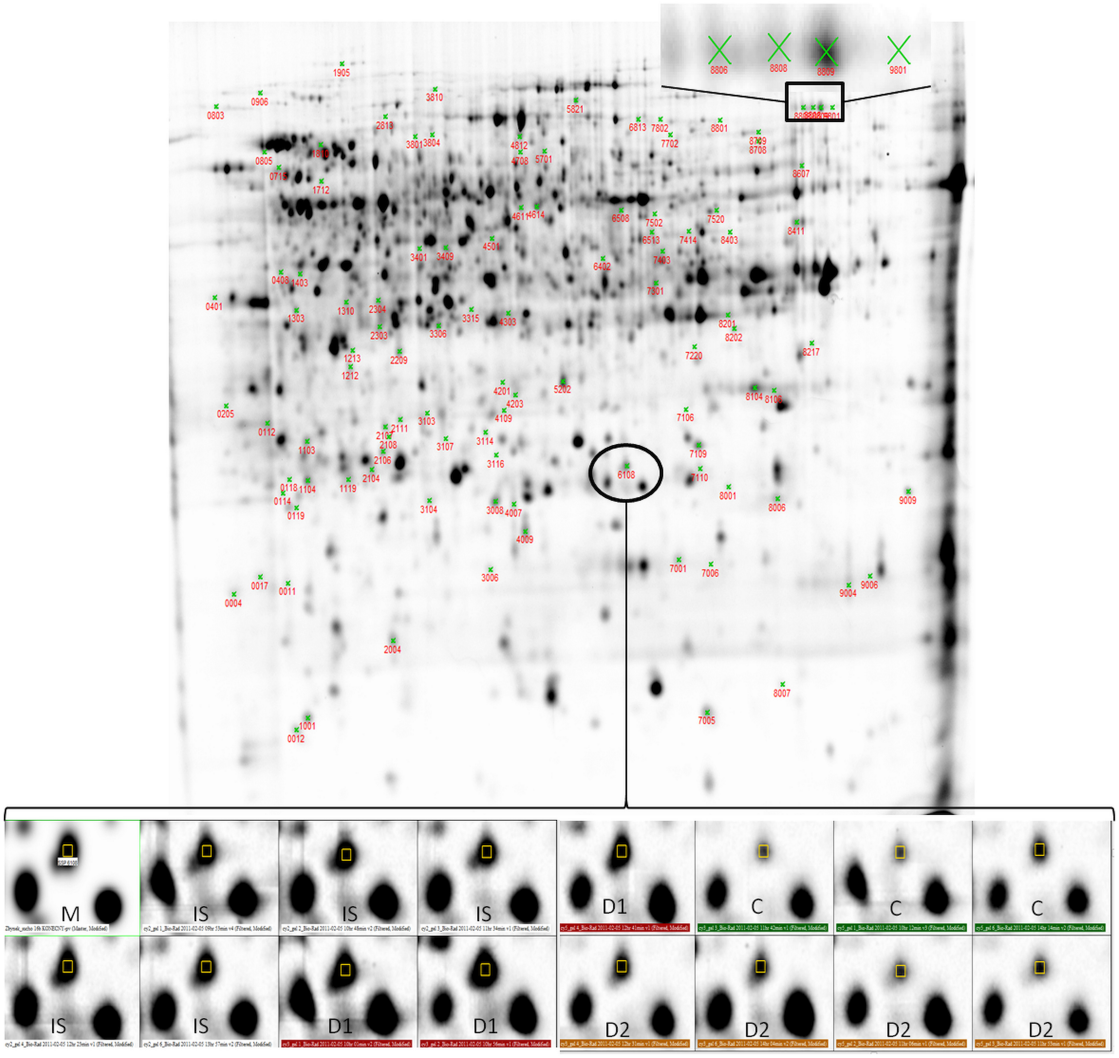
**Representative 2D-DIGE gel (Cy2-labeled pooled sample mixture used as an internal standard) showing 99 differentially abundant protein spots (protein spots revealing at least 2-fold change in relative abundance between control and drought-treated plants) selected for protein identification**.

**Figure 3:**
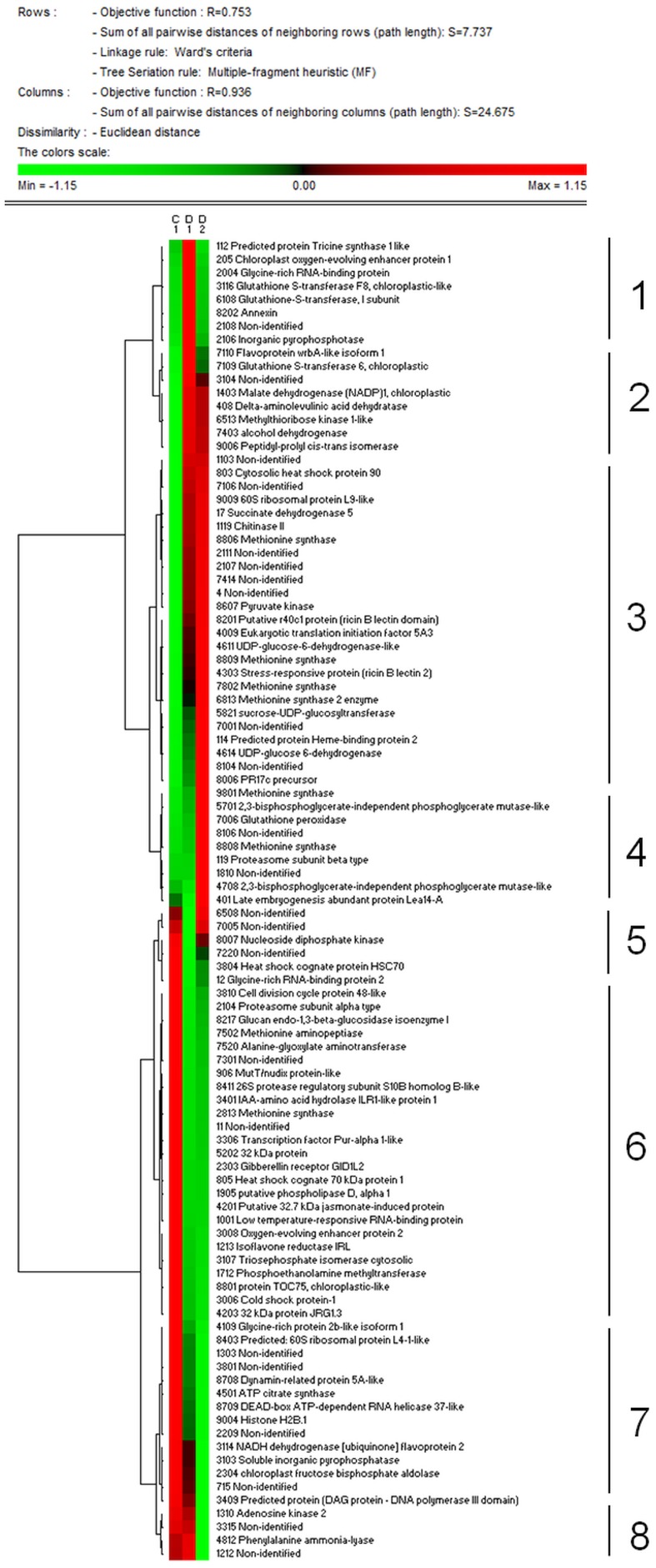
**Results of cluster analysis of 99 protein spots selected for protein identification**. Cluster analysis shows patterns of protein spot relative abundance in samples grown under control (C), drought 1 (D1) and drought 2 (D2) conditions. Euclidean distance and Ward's minimum criteria were applied for the analysis.

**Figure 4:**
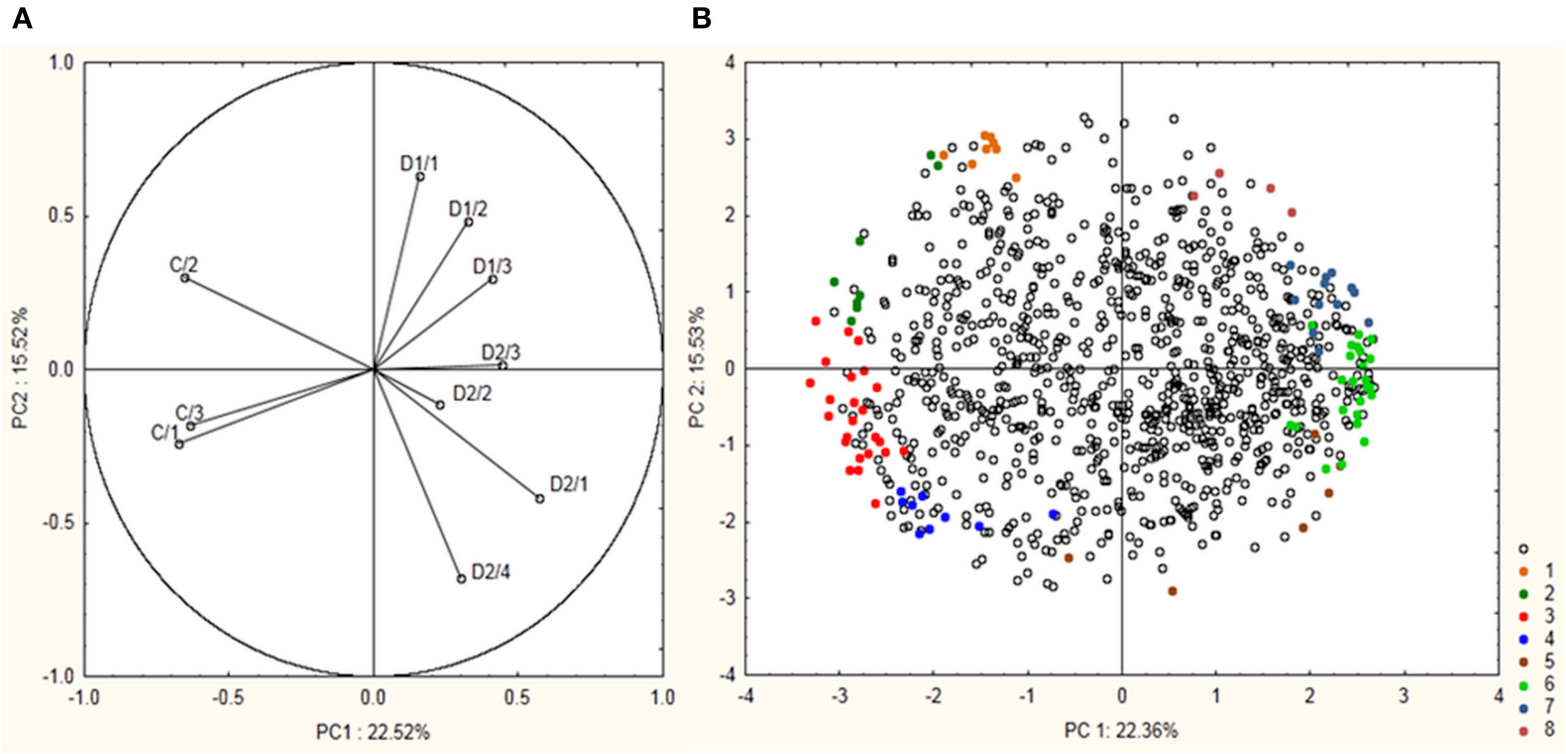
**Results of PCA analysis showing a position of 99 protein spots revealing significant differences between experiment variants and selected for protein identification. (A)** Position of the individual samples (control—C1–C3; drought 1—D1/1–D1/3; drought 2—D2/1–D2/4) are indicated. **(B)** Position of the individual protein spots are indicated. Colored spots 1–8 indicate 99 selected protein spots with their indicated cluster positions according to cluster analysis.

Total proteome analysis of barley crowns using the 2D-DIGE approach has led to detection of 1004 distinct protein spots thorough all gels in experiments (matched and normalized) in the pI range 4–7 (Figure 2, details in Supplementary Data). Quantitative analysis of protein spot density (protein spot relative accumulation) has led to detection of 105 protein spots (spots of interest), revealing significant quantitative differences between the treatments (more than two-fold change at 0.05 level); the spots were selected for MALDI-TOF/TOF protein identification. Eighty-two spots of interest were successfully identified. However, 6 spots (118, 1104, 4007, 6402, 7702, and 8001) showed double identification in the same spots; therefore they were excluded from quantitative analyses. Cluster analysis of the spots of interest revealed eight different patterns of quantitative changes between the three treatments (Figure 3). Principal component analysis (PCA) of all the matched protein spots revealed a clear distinction between the three treatments (C, D1, D2), with a prominent difference between the C and drought treatments (Figure 4A). Protein spots of interest are placed in the distant parts of the PCA protein spot area of protein relative accumulation (Figure 4B). The sum of standard deviations of density of the protein spots under C, D1, and D2 conditions showed different variabilities in the spot density of spots of interest (0.19, 0.17, 0.24, respectively), and in the density of all matched and normalized spots (2.06, 0.68, 1.05, respectively).

**Figure 5:**
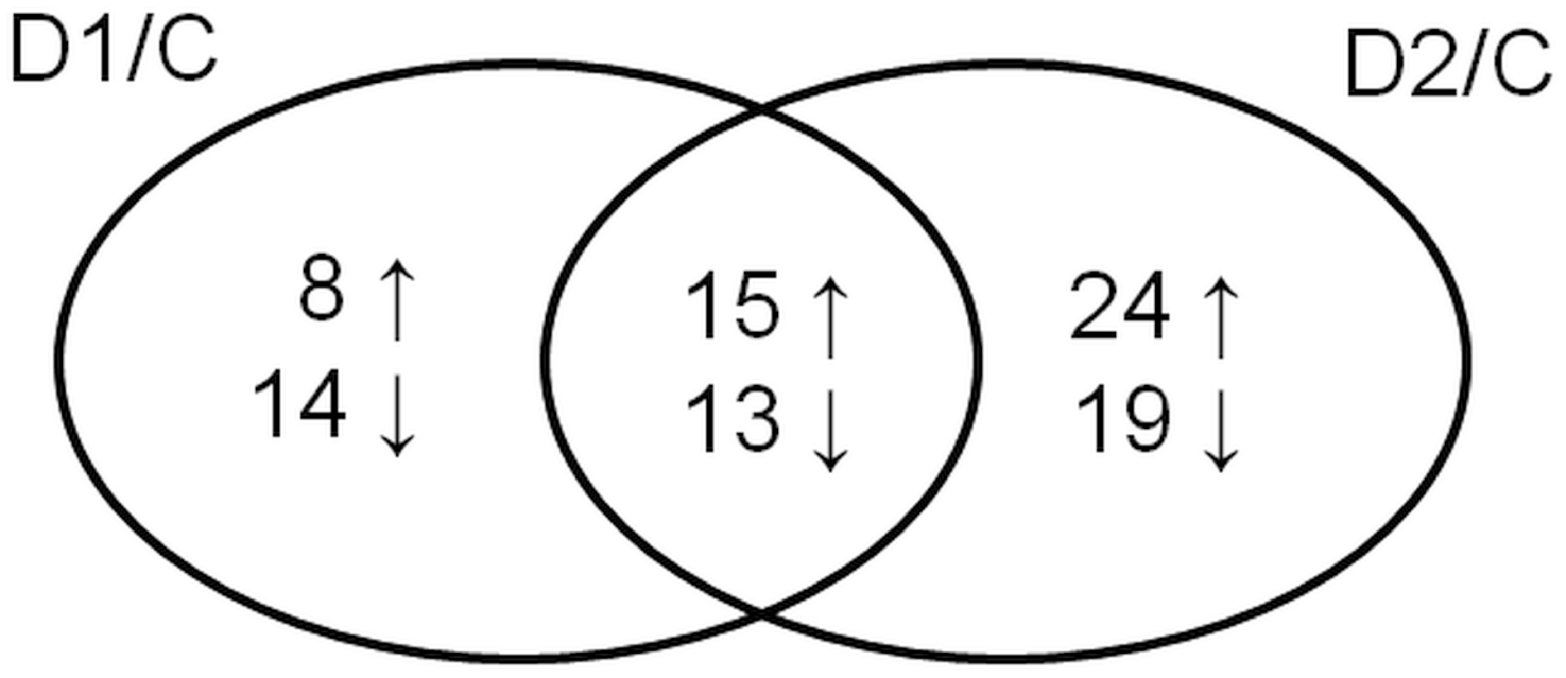
**Venn diagram showing protein spots revealing a significant increase (↑) or a decrease (↓) in drought-treated samples with respect to control ones (D1/C; D2/C)**.

Cluster analysis revealed the presence of 8 clusters based on the differential pattern of protein abundance with respect to the individual treatments (C, D1, D2). Cluster 1 includes proteins with the highest abundance at D1 with respect to the C and D2 treatments; clusters 2 and 3 encompass proteins revealing an increase under drought with respect to the C; cluster 4 includes proteins revealing an enhanced abundance at D2 with respect to D1 and the C; cluster 5 encompasses proteins revealing a decreased abundance at D1 with respect to D2 and C conditions; cluster 6 includes proteins revealing a decrease under both drought treatments with respect to the C; and clusters 7 and 8 encompass proteins revealing a decrease at D2 with respect to D1 and C conditions. A Venn diagram shows that: 8 proteins reveal an increase, and 14 proteins reveal a decrease, specifically in ratio D1/C; further, that 24 proteins reveal an increase, and 19 proteins reveal a decrease, specifically in ratio D2/C; while 15 proteins and 13 proteins are increased and decreased, respectively, under both drought treatments with respect to the C (Figure 5).

**Figure 6:**
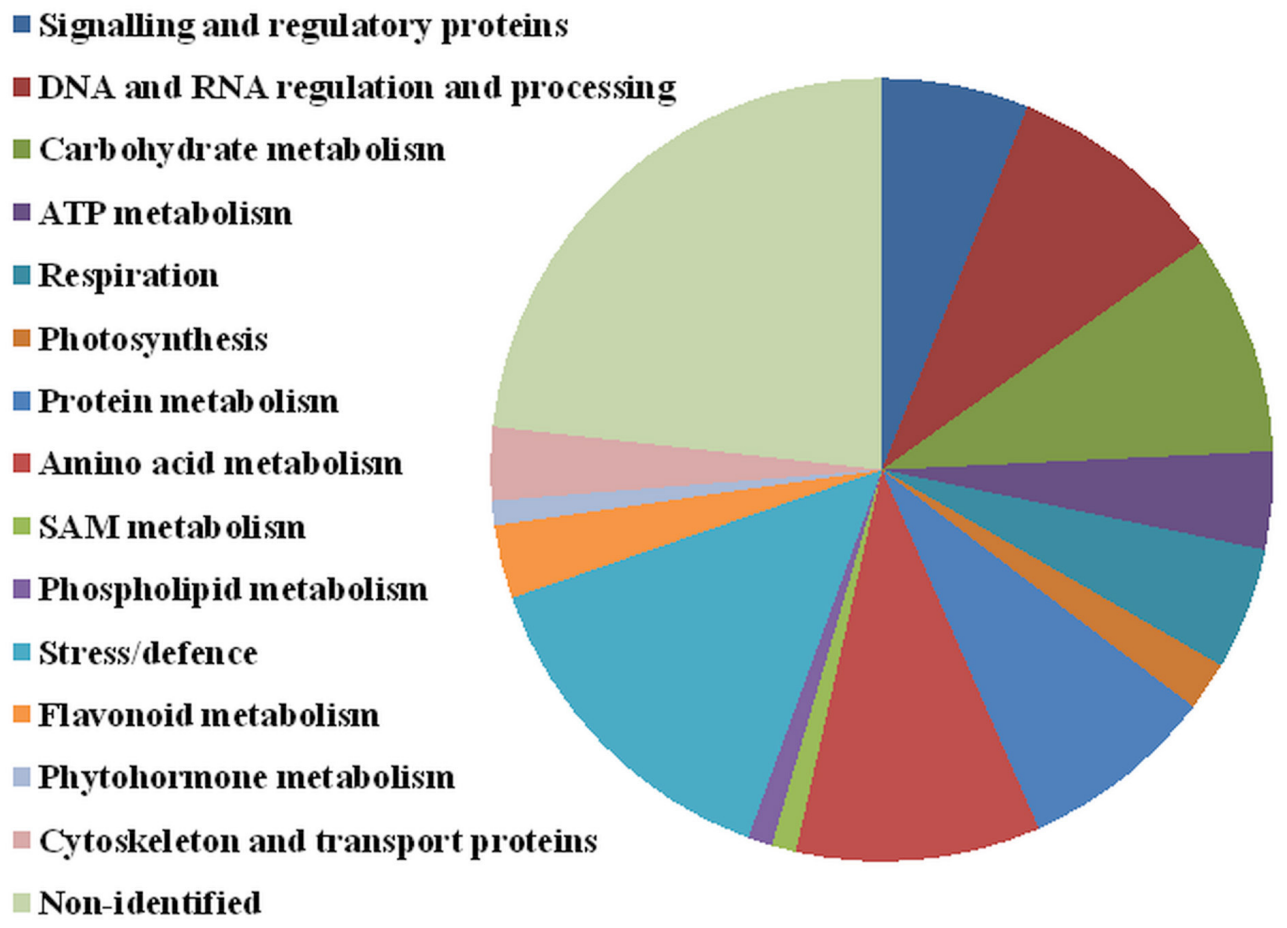
**Protein functional annotation**. Selected 99 protein spots were categorized into the following functional categories: signaling and regulatory proteins (6 spots), DNA and RNA regulation and processing (9 spots), carbohydrate metabolism (9 spots), ATP metabolism (4 spots), respiration (5 spots), photosynthesis (2 spots), protein metabolism (8 spots), amino acid metabolism (10 spots), SAM metabolism (1 spot), phospholipid metabolism (1 spot), stress and defense (14 spots), flavonoid metabolism (3 spots), phytohormone metabolism (1), cytoskeleton and transport (3 spots), and non-identified (23 spots).

Significant differences between D2 and D1 in 27 spots of interest were also found (Table 1, details in Supplementary Data). An increase in accumulation was shown in 15 spots of interest (9 were identified). A decrease in accumulation was revealed in 12 spots of interest (9 were identified). Four protein spots (4614—UDP-glucose 6-dehydrogenase, 7006—glutathione peroxidase, and non-identified spots 7001 and 8104) revealed a continuous significant increase between C, D1, and D2 treatments (in all ratios). No continuous significant decrease was observed in the data set obtained. Seventy-six distinct protein spots were identified as 68 distinct proteins, distributed in 15 major functional categories regarding biological processes (Figure 6, Table 1) including: signaling and regulatory proteins (6 spots), proteins involved in regulation of DNA and RNA activity and processing (9 spots), cytoskeleton and transport proteins (3 spots), proteins involved in energy metabolism including carbohydrate metabolism (9 spots), ATP metabolism (4 spots), respiration (5 spots), and photosynthesis (2 spots), proteins involved in amino acid metabolism (10 spots), protein metabolism (8 spots), S-adenosylmethionine (SAM) metabolism (1 spot), flavonoid metabolism (3 spots), phospholipid metabolism (1 spot), phytohormone metabolism (1 spot), stress and defense responses (14 spots). Four proteins were identified in multiple proteins spots (putative 32.7 kDa jasmonate-induced protein—4201, 5202; UDP-glucose 6-dehydrogenase—4611, 4614; 2,3-bisphosphoglycerate-independent phosphoglycerate mutase-like—4708, 5701; methionine synthase—2813, 7802, 8806, 8808, 8809, 9801). A complete GO annotation (Gene Ontology database) regarding the three GO criteria (cellular localization, molecular function, and biological process) of the identified protein spots and detailed MS/MS analysis is provided in the Supplementary Data.

**Table 1:** **A list of 99 differentially abundant protein spots selected for protein identification**.

**SSP**	**Protein name**	**GI number**	**Organism**	**pI/MW (kD) theor**	**pI/MW (kD) exp**	**Clus-ter**	**Significant ratio**	**Score/E-Value/QM/NP**
**SIGNALING/REGULATORY PROTEINS**								
1905	Putative phospholipase D alpha 1 (Fragment)	209944121	*Triticum monococcum*	5.4/61.744	5.6/300	6	1↓, 2↓, 4↓	143/5.8e-009/21/3
2303	^*^Gibberellin receptor GID1L2	326502616	*Hordeum vulgare*	5.46/36.67	5.72/45.31	6	1↓, 2↓, 4↓	911/9.2e-086/24/9
3810	^*^Cell division cycle protein 48-like	326492542	*Hordeum vulgare*	5.04/65.47	6/90.61	6	1↓	263/5.8e-021/26/5
4201	Putative 32.7 kDa jasmonate-induced protein	1167955	*Hordeum vulgare*	5.97/32.67	6.25/36.05	6	1↓, 2↓, 4↓	177/2.3e-012/19/4
4203	32 kDa protein JRG1.3	2465430	*Hordeum vulgare*	6.06/32.87	6.3/34.83	6	1↓, 2↓, 4↓	286/2.9e-023/17/6
5202	32.7 kDa jasmonate-induced protein	1167955	*Hordeum vulgare*	5.97/32.67	6.51/35.87	6	1↓, 2↓, 4↓	570/1.2e-051 /29/9
**DNA AND RNA REGULATION AND PROCESSING**								
12	^*^Glycine-rich RNA-binding protein 2	151426183	*Hordeum vulgare*	7.88/16.57	5.28/12.13	5	1↓	849/6.6e-081/25/9
906	^*^MutT/nudix protein-like	218191732	*Oryza sativa*	4.99/87.43	5.2/95	6	1↓, 2↓, 4↓	82/0.0082/15/2
1001	^*^Low temperature-responsive RNA-binding protein	326487468	*Hordeum vulgare*	5.45/15.78	5.34/12.63	6	1↓, 2↓, 4↓	667/2.3e-061/21/7
2004	^*^Glycine rich protein, RNA binding protein	326493798	*Hordeum vulgare*	5.41/16.87	5.75/1617	1	1↓, 3↓	756/2.9e-070/17/5
3306	^*^Transcription factor Pur-alpha 1-like	326532740	*Hordeum vulgare*	5.59/33.12	5.99/45.19	6	1↓, 4↓	608/1.8e-055/26/8
3409	^*^Predicted protein: DAG protein (DNA polymerase III domain)	326510151	*Hordeum vulgare*	8.64/45.66	6.02/59.24	7	2↓	384/4.6e-033/19/6
4109	^*^Glycine-rich protein 2b-like isoform 1	326502466	*Hordeum vulgare*	5.95/24.57	6.25/33.28	7	1↓, 2↓, 4↓	460/1.2e-040/19/4
8709	^*^DEAD-box ATP-dependent RNA helicase 37-like	357125045	*Brachypodium distachyon*	8.24/67.36	7.4/79	7	2↓	228/1.8e-017/20/4
9004	Histone H2B.1	122022	*Triticum aestivum*	10/16.3	7.74/17.4	7	2↓	389/1.5e-033/17/4
**CARBOHYDRATE METABOLISM**								
2304	Chloroplast fructose-bisphosphate aldolase	223018643	*Triticum aestivum*	5.94/42	5.73/50.24	7	2↓	98/0.00018/19/3
3107	Triosephosphate isomerase cytosolic	2507469	*Hordeum vulgare*	5.39/26.74	5.99/30.17	6	2↓	115/3.7e-006/17/2
4611	^*^UDP-glucose 6-dehydrogenase-like	326493270	*Hordeum vulgare*	5.9/52.7	6.35/66.75	3	2↑	803/5.8e-075/25/6
4614	^*^UDP-glucose 6-dehydrogenase	326493270	*Hordeum vulgare*	5.9/52.7	6.42/66.7	3	1↑, 2↑, 3↑, 4↑	173/5.8e-012/13/4
4708	^*^2,3-bisphosphoglycerate-independent phosphoglycerate mutase-like	326509673	*Hordeum vulgare*	5.8/62.89	6.35/80.67	4	2↑, 3↑	141/9.2e-009/19/2
5701	^*^2,3-bisphosphoglycerate-independent phosphoglycerate mutase-like	326509673	*Hordeum vulgare*	5.8/62.89	6.46/80.15	4	2↑, 3↑, 4↑	466/2.9e-041/35/6
5821	^*^Sucrose-UDP glucosyltransferase 1	326514918	*Hordeum vulgare*	5.85/92.2	6.6/87.83	3	2↑	734/4.6e-068/49/8
8217	Glucan endo-1,3-beta-glucosidase isoenzyme I	3037080	*Hordeum vulgare*	7.7/32.96	7.63/38	6	1↓	870/1.2e-081/31/10
8607	^*^Pyruvate kinase	326495152	*Hordeum vulgare*	7.51/55.45	7.59/70	3	1↑, 2↑, 4↑	412/7.3e-036/30/5
**ATP METABOLISM**								
1310	^*^Adenosine kinase 2	151420669	*Hordeum vulgare*	5.07/37.17	5.58/50.37	8	2↓	84/0.00021/9/1
2106	Inorganic pyrophosphatase	388271212	*Triticum aestivum*	5.41/24.28	5.72/29.45	1	1↑, 3↓	298/1.8e-024/23/5
3103	Soluble inorganic pyrophosphatase	4033417	*Hordeum vulgare*	5.85/24	5.92/33.25	7	3↓	101/9.2e-005/10/1
8007	^*^Nucleoside diphosphate kinase	326511160	*Hordeum vulgare*	9.13/26.5	7.45/12.46	5	1↓, 3↑	150/1.2e-009/18/4
**RESPIRATION**								
17	^*^Succinate dehydrogenase 5	18652408	*Hordeum vulgare*	6.92/26.1	5.15/20.61	3	2↑, 4↑	341/9.2e-029/16/4
1403	^*^Malate dehydrogenase [NADP] 1, chloroplastic	326520796	*Hordeum vulgare*	5.89/46.92	5.37/55.62	2	1↑, 2↑, 4↑	265/3.7e-021/14/4
3114	^*^NADH dehydrogenase [ubiquinone] flavoprotein 2	326502384	*Hordeum vulgare*	7.56/30.11	6.17/30.67	7	2↓	350/1.2e-029/26/6
4501	^*^ATP-citrate synthase	326507652	*Hordeum vulgare*	5.55/46.79	6.22/60.26	7	2↓	426/2.9e-037/25/5
7403	Alcohol dehydrogenase	119388733	*Triticum dicoccoides*	6.28/41	6.96/55.36	2	1↑, 2↑, 4↑	402/7.3e-035/20/5
**PHOTOSYNTHESIS**								
205	Chloroplast oxygen-evolving enhancer protein 1	147945622	*Leymus chinensis*	6.09/34.5	5.09/35.52	1	1↑, 3↓	190/1.2e-013/17/3
3008	Oxygen-evolving enhancer protein 2	131394	*Triticum aestivum*	5.95/20	6.19/24.48	6	2↓	303/5.8e-025/25/5
**PROTEIN METABOLISM**								
119	^*^Proteasome subunit beta type	326488821	*Hordeum vulgare*	5.16/22.77	5.3/24.8	4	2↑, 3↓	250/1.2e-019/10/2
2104	20S Proteasome subunit alpha type	115447473	*Oryza sativa*	5.36/25.84	5.68/27.53	6	1↓	443/5.8e-039/16/4
4009	Eukaryotic translation initiation factor 5A3	74049040	*Triticum aestivum*	5.76/17.38	6.33/22.48	3	2↑, 4↑	244/4.6e-019/15/3
7502	Methionine aminopeptidase	301508493	*Hordeum vulgare*	6.58/43.37	6.92/62.49	6	1↓, 2↓, 4↓	273/5.8e-022/31/7
8403	^*^PREDICTED: 60S ribosomal protein L4-1-like	326488173	*Hordeum vulgare*	10.58/44.18	7.27/57.29	7	2↓, 4↓	388/1.8e-033/31/6
8411	26S protease regulatory subunit S10B homolog B-like	357123904	*Brachypodium distachyon*	7.03/44.75	7.56/57.63	6	1↓, 4↓	493/5.8e-044/31/7
9006	Peptidyl-prolyl cis-trans isomerase	385145559	*Oryza brachyantha*	8.27/18.38	7.8/17.7	2	1↑, 2↑, 4↑	136/2.9e-008/9/3
9009	60S ribosomal protein L9-like	357110922	*Brachypodium distachyon*	9.86/21.32	7.9/23.5	3	2↑, 4↑	433/5.8e-038/23/3
**AMINO ACID METABOLISM**								
408	^*^Delta-aminolevulinic acid dehydratase	326490676	*Hordeum vulgare*	5.72/46.1	5.27/55.68	2	2↑, 4↑	246/2.9e-019/19/3
2813	Methionine synthase	50897038	*Hordeum vulgare*	5.67/84.5	5.78/87.6	6	1↓, 2↓, 4↓	626/2.9e-057/34/7
4812	Phenylalanine ammonia-lyase	326526355	*Hordeum vulgare*	5.89/75.62	6.35/85.17	8	3↓	734/4.6e-068/41/9
6813	^*^Methionine synthase 2 enzyme	326513870	*Hordeum vulgare*	5.82/84.43	6.87/85.62	3	2↑, 4↑	135/3.7e-008/25/3
7520	^*^Alanine-glyoxylate aminotransferase	326496042	*Hordeum vulgare*	7.63/51.77	7.22/71.67	6	1↓, 4↓	228/1.8e-017/22/4
7802	Methionine synthase	50897038	*Hordeum vulgare*	5.67/84.51	6.96/85.51	3	1↑, 2↑, 4↑	197/2.3e-014/23/4
8806	Methionine synthase	50897038	*Hordeum vulgare*	5.67/84.5	7.6/85.28	3	1↑, 2↑, 4↑	366/2.9e-031/26/4
8808	Methionine synthase	50897038	*Hordeum vulgare*	5.67/84.5	7.64/85.15	4	2↑, 3↑, 4↑	1160/1.2e-110/42/9
8809	Methionine synthase	50897038	*Hordeum vulgare*	5.67/84.5	7.68/85	3	2↑, 4↑	419/1.5e-036/27/7
9801	Methionine synthase	50897038	*Hordeum vulgare*	5.67/84.5	7.7/85	4	2↑	488/1.8e-043/27/5
**SAM METABOLISM**								
6513	^*^Methylthioribose kinase 1-like	326498609	*Hordeum vulgare*	6.15/46.75	6.91/59.62	2	1↑, 2↑, 4↑	421/9.2e-037/29/7
**PHOSPHOLIPID METABOLISM**								
1712	Phosphoethanolamine methyltransferase	17887465	*Triticum aestivum*	5.21/56.85	5.49/76.71	6	2↓, 4↓	514/4.6e-046/23/6
**STRESS AND DEFENS**								
114	^*^Heme-binding protein 2	326488153	*Hordeum vulgare*	5.26/23.93	5.23/25.97	3	2↑, 4↑	260/1.2e-020/16/6
401	^*^Late embryogenesis abundant protein Lea14-A	326528557	*Hordeum vulgare*	4.94/36.25	5.06/51.74	4	3↑	583/5.8e-053/28/7
803	^*^Cytosolic heat shock protein 90	32765549	*Hordeum vulgare*	4.95/80.42	5.1/90	3	1↑, 2↑, 4↑	180/1.2e-012/28/5
805	^*^Heat shock cognate protein HSC70	326506132	*Hordeum vulgare*	5.07/71.12	5.21/84.55	6	1↓, 2↓, 4↓	897/2.3e-084/41/9
1119	Chitinase II	9501334	*Hordeum vulgare*	9.12/27.13	5.57/26.99	3	2↑	82/0.007/6/2
3006	^*^Cold shock protein-1	326510343	*Hordeum vulgare*	5.78/18.17	6.17/20.01	6	1↓, 2↓, 4↓	435/3.7e-038/15/5
3116	^*^Glutathione S-transferase F8, chloroplastic-like	326533368	*Hordeum vulgare*	5.78/25.64	6.21/28.31	1	1↑, 3↓	96/0.00031/8/2
3804	Heat shock cognate protein HSC70	2655420	*Brassica napus*	5.07/70.77	5.97/84.85	5	1↓, 4↓	136/2.9e-008/23/1
4303	^*^Stress-responsive protein (ricin B lectin 2 domain)	326517467	*Hordeum vulgare*	5.71/35.62	6.28/46.39	3	2↑, 4↑	449/1.5e-039/24/6
6108	Glutathione-S-transferase, I subunit	21212950	*Hordeum vulgare*	5.86/23.48	6.77/26.55	1	1↑	494/4.6e-044/20/8
7006	Glutathione peroxidase	6179604	*Hordeum vulgare*	6.72/18.26	7.14/19.03	4	1↑, 2↑, 3↑, 4↑	336/2.9e-028/17/4
7109	^*^Glutathione S-transferase 6, chloroplastic	326529393	*Hordeum vulgare*	6.45/12.38	7.1/25.65	2	1↑, 4↑	249/1.5e-019/19/6
8006	^*^PR17c precursor	2266666	*Hordeum vulgare*	8.56/24.23	7.45/23.46	3	2↑, 3↑, 4↑	271/9.2e-022/21/5
8201	^*^Putative r40c1 protein (ricin B lectin domain)	326497973	*Hordeum vulgare*	6.27/38.74	7.26/42.78	3	2↑	314/4.6e-026/27/5
**FLAVONOID METABOLISM**								
112	^*^PREDICTED: tricin synthase 1-like	326494234	*Hordeum vulgare*	4.92/27.1	5.18/33.28	1	3↓	821/9.2e-077/30/7
1213	^*^Isoflavone reductase IRL	151421343	*Hordeum vulgare*	5.33/33.25	5.61/42.33	6	1↓, 2↓, 4↓	450/5.2e-041/21/6
7110	^*^Flavoprotein wrbA-like isoform 1	326516502	*Hordeum vulgare*	6.21/21.78	7.11/25.36	2	1↑, 4↑	525/3.7e-047/24/8
**PHYTOHORMONE METABOLISM**								
3401	^*^IAA-amino acid hydrolase ILR1-like protein 1	326491655	*Hordeum vulgare*	5.55/47.75	5.91/59.27	6	1↓	280/1.2e-022/25/4
**CYTOSKELETON AND TRANSPORT**								
8202	^*^Annexin	326528789	*Hordeum vulgare*	6.52/35.25	7.29/40.9	1	1↑, 3↓	795/3.7e-074/42/9
8708	^*^Dynamin-related protein 5A-like protein	115465357	*Oryza sativa*	7.65/68.68	7.4/76.62	7	2↓, 3↓, 4↓	524/4.6e-047/26/7
8801	^*^Protein TOC75, chloroplastic-like	326506018	*Hordeum vulgare*	7.95/86.92	7.24/85	6	2↓, 4↓	257/2.3e-020/37/8
**NON-IDENTIFIED**								
4	Non-identified				5.1/19.53	3	2↑, 4↑	
11	Non-identified				5.25/20.09	6	1↓, 4↓	
715	Non-identified				5.29/80.08	7	2↓, 4↓	
1103	Non-identified				5.37/31	3	1↑, 2↑, 4↑	
1212	Non-identified				5.59/40.18	8	2↓, 3↓	
1303	Non-identified				5.34/49.2	7	2↓	
1810	Non-identified				5.49/85.45	4	2↑, 3↑	
2107	Non-identified				5.74/32.1	3	1↑, 2↑, 4↑	
2108	Non-identified				5.76/30.97	1	3↓	
2111	Non-identified				5.8/32.64	3	2↑, 4↑	
2209	Non-identified				5.81/41.57	7	2↓	
3104	Non-identified				5.93/24.73	2	1↑, 2↑, 4↑	
3315	Non-identified				6.12/47.68	8	2↓, 3↓	
3801	Non-identified				5.91/85.34	7	2↓	
6508	Non-identified				6.78/65.2	5	3↑	
7001	Non-identified				6.99/19.56	3	1↑, 2↑, 3↑, 4↑	
7005	Non-identified				7.1/11.44	5	1↓, 3↑	
7106	Non-identified				7.04/31.34	3	2↑, 4↑	
7220	Non-identified				7.09/39.01	5	1↓, 3↑, 4↓	
7301	Non-identified				6.92/49.52	6	1↓, 4↓	
7414	Non-identified				7.08/58.44	3	2↑	
8104	Non-identified				7.36/33.22	3	1↑, 2↑, 3↑, 4↑	
8106	Non-identified				7.44/32.84	4	2↑, 4↑	

The data on each protein include protein ssp number, protein name (^*^) in the case of uncharacterized predicted proteins the name came from the best BLAST result against NCBI database—www.ncbi.nlm.nih.gov) and its GI number from NCBI database (www.ncbi.nlm.nih.gov), name of organism from which the given protein sequence (GI number) comes from NCBI database, data on protein spot basic characteristics—theoretical pI and MW values determined from the sequence, experimental pI and MW values determined from the protein spot position on 2D-DIGE gel, cluster number determined by cluster analysis, significant ratio of the changes in protein spot relative abundance between control and drought-treated samples (see below), and basic parameters related to protein spot MALDI-TOF/TOF identification. Regarding protein identification, ^*^means that the protein GI sequence points to a predicted protein with unknown function and that the protein possible identity was determined by a PBLAST search against NCBInr database.↑, significant increase in ratio upon drought than in control; ↓, significant decrease in ratio upon drought than in control; 1, D1/Control; 2, D2/Control; 3, D2/D1; 4, Drought/Control; exp, experimental; E-Value, Expectation value; NP, number of sequenced peptides by MS/MS fragmentation; QM, matched peptides; Score, MS Score; theor, theoretical.

(*) *in the case of uncharacterized predicted proteins the protein name came from the best BLAST result against NCBI database—www.ncbi.nlm.nih.gov, for detail see Supplementary Data*.

## Discussion

### The effect of drought compared to control

Two different drought intensities (35 and 30% of SWC; D1 and D2, respectively) were studied in the experiment. Drought induces profound alterations in plant metabolism directed toward an adjustment of plant cells to dehydration. Determination of physiological parameters WSD, OP, and Δ^13^C revealed significant dehydration and limited stomatal openness (limited CO_2_ availability) in drought-treated barley plants vs. controls. Similarly, an enhanced accumulation of dehydrin protein DHN5 on the immunoblots indicates cellular dehydration. Interestingly, standard deviation in the physiological parameters and spot density indicated some trends in the samples. The least difference in variability of the data found in D1 samples could indicate a functional stress response of plants (slower growth and development, accumulation of stress, and defense proteins and metabolites). The higher variability in the control condition could be the result of faster growth and development of plants under optimal watering, while the population of individual plants could be slightly differentiated in the same biological repetition. The higher variability in D2 could be related to more severe water condition of the plants, where the damage could influence the physiological and quantitative proteomics data. What could happen with the sample if in some part of the plant a higher ratio of a senescence process or cell death occurred? For example, density analysis of spot 7109 (GST6, cluster 2; Supplementary Data) revealed that after a high increase in D1, the accumulation was decreased in D2. However, in a detailed view, half of the D2 samples accumulated at similar levels as in D1; and in the other half at a similar level as in the C. Therefore, the variability in the data set could represent an additional explanation of plant status (stress response and plant damage) and the obtained proteome results, which are discussed in the following parts of the text.

The qualitative analysis of proteome not only significantly distinguished spots between control and drought conditions, but also differences (27 spots) between both drought treatments were found. Some spots identified as one protein (e.g., 5 spots of methionine synthase in clusters 3, 4, and 6) revealed different abundance between treatments and thereby were placed to different clusters. This could indicate different functionality of the isoforms and/or post-translational modifications under varying drought conditions and demonstrates one of the main advantages of the gel-based method compared to the gel-free approach. For example, the isoform analysis was shown by Erban and Hubert (2015) for zymogens and active-enzyme forms of house dust mite fecal allergens.

In comparison to previous proteome studies on barley's response to drought treatment, in this study, more proteins were found and identified (even for more robust protein accumulation between treatments = two-fold change). Wendelboe-Nelson and Morris (2012) identified 24 leaf and 45 root differentially accumulated proteins (however, the roots were cultivated under different conditions than the leaves) between the drought sensitive European malting barley Golden Promise (GP), and the Iraqi Basrah barley adapted to hot and dry conditions. Only four proteins from the leaf tissue (HSP70, OEE1 and 2, and methionine synthase) were the same as in our study. GP showed a lower expression and/or accumulation of constitutively present proteins, which could be connected to the slower response of GP to stress, compared to Basrah. According to the results of Wendelboe-Nelson and Morris (2012), our cv. Amulet showed a similar accumulation pattern as a sensitive GP. However, the authors did not show any detailed information about soil water content between genotypes (e.g., caused by different rates of transpiration), and did not carefully take into account their possible different response in biomass allocations (and thus their real drought adaptability). Ashoub et al. (2013) found about 22 accumulated and 6 down-accumulated proteins between tolerant (#15,141) and sensitive (#15,163) cultivars. After 5 days, pot soil field capacity drops to 10%; this evokes very sandy soil, quick, and deep stress with reduced genotype-based acclimation ability. Compared to Ashoub et al. (2013), only 3 proteins (methionine synthase, HSP90, and HSP70) were identified also in our study. Ghabooli et al. (2013) found 62 protein spots (only 45 was identified) with significant differences between *Piriformospora indica*-colonized GP plants compared with non-inoculated plants in response to drought stress (14 days; 25% field capacity). Compared to our study, only OEE1, peptidyl-prolyl cis-trans isomerase, and 60S ribosomal protein were found in Ghabooli et al. (2013). Kausar et al. (2013) identified 24 protein spots extracted from shoots in drought-sensitive Pakistani genotype 004186 and 19 spots in drought-tolerant Pakistani genotype 004223 after only 3 days of treatment. The identifications shared with our study included only protein spots identified as malate dehydrogenase, HSP70, OEE1, OEE2, methionine synthase, and glutathione transferase (GST). We found an analogous protein accumulation to patterns found in sensitive genotypes in all studies mentioned above (for details, see the text below).

In the paragraphs below, the proteins identified are briefly discussed with respect to their biological functions:

Proteins involved in signaling and regulatory processes, phospholipid metabolism:

The accumulations of all of proteins in this functional group decreased under drought conditions, and belong in cluster 6. Generally, the decrease could be explained by a reduction of plant growth and development under drought conditions.

Putative phospholipase D, alpha 1 (ssp 1905) catalyzes the cleavage of phospholipids, leading to formation of phosphatidic acid (PA) and other small molecules that can act as signals.

Proteins (ssp 4201, 4203, and 5202) were identified as jasmonate-regulated lectins. Several lectins have been reported to accumulate in cereal crown tissues, where the shoot apex is located, and to affect shoot apex development. For example, an accumulation of lectin VER2 was reported in cold-treated wheat crown tissue until vernalization (Rinalducci et al., 2011; Kosová et al., 2013). Therefore, in our study, the observed results (i.e., a decrease under drought) in these proteins indicated that the proteins belong into the several lectins with some stimulating role in plant development contrary to VER2 lectin in plants under cold treatment found in Rinalducci et al. (2011) and Kosová et al. (2013).

Gibberellin receptor GID1L2 (ssp 2303) is a part of the gibberellins (GAs) perception process (Ueguchi-Tanaka et al., 2007). A decrease in ssp 2303 relative abundance under both drought treatments, with respect to the control, corresponds well with the adverse effects of stress on plant growth and development.

Protein cdc48 (ssp3810) is involved in the cell division process, and is known to be downregulated in differentiated cell types. Up to now, no evidence of such protein identification was found in studies on plant abiotic stress response. However, Skadsen et al. (2000) found also decrease of cdc48 mRNA after inoculation of barley spikes with *Fusarium graminearum*.

Proteins involved in DNA and RNA regulatory processes:

Generally, the proteins involved in DNA and RNA regulatory processes have a role in plant development or growth and are decreased under drought.

MutT/nudix protein (ssp 906; cluster 6) belongs to the family of nucleoside diphosphate hydrolases, which are involved in the repair of DNA during replication. A revealed decrease in accumulation could indicate a reduced speed of replication (i.e., slower plant growth and development).

Histone H2B.1 (ssp 9004; cluster 7) belongs within the histone group. Several nucleosomal histones (histone H2A.1, histone H2B.10, histone H3.2) were found to be altered (increased or decreased) in germinating durum wheat seedlings upon salt stress (Fercha et al., 2013).

Changes in glycine-rich RNA binding proteins (ssp 12, cluster 5; 2004, cluster 1) were reported also in wheat upon cold (Rinalducci et al., 2011; Kosová et al., 2013). The members of the glycine-rich RNA-binding protein family are known to regulate RNA processing, transport, and to reveal regulatory functions.

Transcription factor Pur-alpha-1 (ssp 3306, cluster 6) is involved in the initiation of nuclear DNA replication. Its decrease during drought may indicate a reduced rate of cell division upon stress conditions. It is in according with a decrease in protein containing DNA polymerase III domain (ssp 3409, cluster 7) accumulation. DNA polymerase III is a prokaryotic DNA polymerase involved in the replication of circular DNA; its homologs are found in plants in mitochondria and plastids (mitochondrial and plastidic DNA polymerases).

DEAD-box ATP-dependent RNA helicase (ssp 8709, cluster 7) was described to regulate mRNA export from nucleus to cytoplasm (i.e., to function as a RNA chaperone). In *Arabidopsis thaliana*, a mutation in the locus encoding DEAD-box RNA helicase has led to enhanced cold induction of CBF2 and its downstream genes including Cor/Lea genes (Gong et al., 2005). According to Wendelboe-Nelson and Morris (2012), who found increase of DEAD box RNA helicase in drought-stressed roots of drought-tolerant Basrah compared to no change in sensitive genotype GP. Taken together, a higher trend in dehydrin accumulation and revealed decrease of this protein is also supporting our idea about Amulet as a sensitive genotype.

### Energy metabolism—ATP metabolism, carbohydrate metabolism, photosynthesis, respiration

Stress factors profoundly affect energy metabolism, since plant adjustment to an altered environment generally means an enhanced need for immediately available energy. Changes in several enzymes involved in ATP metabolism, especially the cleavage of phosphate bonds, were found in our study (adenosine kinase 2—ssp 1310, cluster 8; inorganic pyrophosphatase—ssp 2106, cluster 1, and ssp 3103, cluster 7; nucleoside diphosphate kinase—ssp 8007, cluster 5). An enhanced need for ATP as a universal energy source has been reported in many proteomic studies aimed at plant stress responses, as indicated by the reports on increases in ATP synthase subunits (Vítámvás et al., 2012; Kausar et al., 2013). The major sources of novel ATP molecules represent both processes of anaerobic and aerobic respiration as well as photosynthesis. The anaerobic portion of respiration includes glycolysis. An increased relative abundance of glycolytic enzymes was found in several proteomic studies on stress-treated plants (Vítámvás et al., 2012; Kosová et al., 2013). However, in the present study, a decrease in some glycolytic enzymes: cytosolic triosephosphate isomerase (ssp 3107, cluster 6), and chloroplast fructose bisphosphate aldolase (ssp 2304, cluster 7); and an increase in others: 2,3-bisphosphoglycerate-independent phosphoglycerate mutase-like (ssp 4708, 5701, cluster 4), and pyruvate kinase (ssp 8607, cluster 3) were found under drought, with respect to the controls. A possible explanation of the observed difference could lie in the fact that the samples for proteome analysis were taken from plants exposed to long-term drought treatment, and the plants were fully acclimated to altered conditions without need for extra energy.

Regarding anaerobic respiration, an increase in alcohol dehydrogenase (ssp 7403, cluster 2) was found under drought. Regarding aerobic respiration, a drought-induced decrease in Krebs cycle enzyme ATP-citrate synthase (ssp 4501, cluster 7) and in complex I of respiratory electron transport chain (NADH dehydrogenase [ubiquinone] flavoprotein 2—ssp 3114, cluster 7) was found. In contrast, the levels of other Krebs cycle enzymes—succinate dehydrogenase (ssp 17, cluster 3), and malate dehydrogenase (ssp 1403, cluster 2) were increased upon drought with respect to the controls. These data indicate stress-induced imbalances in aerobic metabolism (imbalances between primary electron transport reactions and secondary enzymatic reactions) and an enhanced risk of ROS formation, which results in the downregulation of aerobic electron transport reactions, and a relative upregulation of alternative anaerobic pathways. Similar results (increase in alcohol dehydrogenase, formate dehydrogenase, aldehyde dehydrogenase) were obtained by Fercha et al. (2014) in salt-treated germinating wheat seedlings indicating the severe impact of drought on the aerobic portion of energy metabolism in our study.

Photosynthesis is known to be very sensitive to several stresses including cold, drought, and salinity. In our study, changes in two components of the oxygen-evolving complex (OEC) were found: proteins OEE1 (PsbO; ssp 205, cluster 1), and OEE2 (PsbP; ssp 3008, cluster 6). These dynamics indicated an increase under the milder drought (D1) with respect to the controls; however, a decrease under the more severe drought (D2). Changes in OEC proteins were found in drought-treated wheat genotypes (intolerant Kukri; tolerant Excalibur, and RAC875; Ford et al., 2011). Changes in OEE1 and OEE2 proteins were frequently found in salt-treated barley (Rasoulnia et al., 2011; Fatehi et al., 2012) and durum wheat (Caruso et al., 2008). Moreover, increase of OEC proteins were found in our previous studies on cold-acclimated wheat (Vítámvás et al., 2012) or barley (Hlaváčková et al., 2013). Additionally, an increase in OEE1 protein was observed in drought-treated barley infected by *Piriformospora indica* (Ghabooli et al., 2013). Wendelboe-Nelson and Morris (2012) have demonstrated a decrease of OEE1 in stressed leaves of the sensitive barley GP, compared to the increase of OEE2 in tolerant Basrah. Kausar et al. (2013) showed an increase in OEE proteins under milder drought and in tolerant plant materials; while also showing a decrease under severe drought or in sensitive plant materials. These findings are in accordance with our findings, and we can postulate Amulet as a sensitive genotype to drought. However, the lack of other photosynthetic proteins corresponded with the material used (crowns are a non-photosynthetic tissue; see (Hlaváčková et al., 2013) for a comparison of crown and leaf proteome); therefore, only two photosynthetic proteins with a difference in protein accumulation were found.

Regarding carbohydrate anabolism, an increased relative abundance of UDP-glucose 6-dehydrogenase (ssp 4611, 4614, cluster 3), and sucrose-UDP-glucosyltransferase (ssp 5821, cluster 3) was found under drought. An increase in UDP-glucose 6-dehydrogenase may indicate enhanced synthesis of pectins and hemicelluloses, as well as the remodeling of cell walls in response to stress. Generally, in our previous studies on cold-acclimated wheat and barley (Vítámvás et al., 2012; Hlaváčková et al., 2013), the decrease of accumulation of the sucrose-UDP-glucosyltransferase and UDP-glucose 6-dehydrogenase was observed. It implicates specific plant response to different abiotic stresses.

### Protein metabolism, amino acid metabolism, SAM metabolism

The stress acclimation process is also associated with significant alterations in protein metabolism, regarding both protein biosynthesis and degradation. Alterations in protein biosynthesis are reflected in the changes of 60S ribosomal proteins L4-1-like (ssp 8403, cluster 7), L9-like (ssp 9009, cluster 3), as well as in eukaryotic translation initiation factor 5A3 (ssp 4009, cluster 3). Alterations in ribosomal proteins are described in several studies that focused on stressed wheat and barley plants (Patterson et al., 2007; Vítámvás et al., 2012; Ghabooli et al., 2013; Fercha et al., 2014; Gharechahi et al., 2014), which indicated an enhanced need for novel proteins during stress acclimation. An increase in eukaryotic translation initiation factor eIF5A3 (ssp 4009) was found under drought with respect to the C. It has been found that eIF5A not only functions as an initiation translation factor, but it can also undergo a post-translational modification of lysine residue to hypusine, and that the stoichiometry of different hypusinated forms of eIF5A can affect a switch between cell proliferation and cell death (Thompson et al., 2004). An increase in eIF5A2 in spring wheat Sandra under cold (with respect to the C), and a relatively enhanced level of eIF5A2 in spring wheat Sandra (with respect to winter wheat Samanta) was found by Kosová et al. (2013).

Protein conformation is regulated by peptidyl-prolyl *cis-trans* isomerase (ssp 9006, cluster 2). Alterations in two isoforms of peptidyl-prolyl *cis-trans* isomerase were also found in barley cv. GP colonized by *Piriformospora indica* when subjected to drought (Ghabooli et al., 2013). An increased rate of protein degradation, associated with alterations in the metabolism of stressed plants, is indicated by a drought-increased level of the proteasome subunit beta type (ssp 119, cluster 4). However, some identified proteasome subunits proteins revealed a decrease in accumulation—i.e., 26S protease regulatory subunit S10B homolog B-like (ssp 8411, cluster 6), and proteasome subunit alpha type (ssp 2104, cluster 6). Proteasomes are involved in the degradation of ubiquitin-tagged proteins. Alterations in proteasome subunits were found in several proteomic studies dealing with abiotic stresses (Rampitsch et al., 2006; Rinalducci et al., 2011; Fercha et al., 2013; Ghabooli et al., 2013).

Aminopeptidases catalyze protein degradation by the hydrolysis of N-terminal amino acid. Methionine aminopeptidase (ssp 7502, cluster 6) was found to be decreased upon both drought treatments with respect to the control. However, leucine aminopeptidase RNAs, proteins, and activities have been found to be increased following drought and wound stress signal systems, such as methyl jasmonate and abscisic acid in tomato (Chao et al., 1999).

Significant alterations were also found in several enzymes involved in amino acid metabolism. It should be noted that amino acids not only form peptides and proteins, but they are also involved in the metabolism of carbon and nitrogen, as well as in the metabolism of several stress-related compounds (e.g., S-adenosylmethionine metabolism, metabolism of phenolic compounds).

Delta-aminolevulinic acid dehydratase (ssp 408) catalyzes the first and rate-limiting step of the conversion of non-protein amino acid delta-aminolevulinic acid to porphyrin molecules, namely chlorophyll. In our results, the cv. Amulet showed an increase of this protein in D2 and D, which is connected to the greater accumulation of Heme-binding protein (ssp 114), and an increase in glutathione S-transferase (ssp 3116, 6108, and 7109). An increase in delta-aminolevulinic acid causes porphyria (Vanhee et al., 2011). All 6 of the identified methionine synthases, except one, showed increased accumulation upon drought. Wendelboe-Nelson and Morris (2012) found an increase in stressed leaves of the tolerant Basrah genotype; while Ashoub et al. (2013) found a non-significant difference between tolerant and sensitive genotypes. On the basis of published results, there are still some questions about the ability of methionine synthase to distinguish tolerant or sensitive genotypes. Methionine synthase catalyzes biosynthesis of methionine, which is not only a protein amino acid, but also a precursor of S-adenosylmethionine (SAM). S-adenosylmethionine, and the S-methylated form of methionine, not only represents a universal methyl donor in plant cells, it also functions as a precursor of several stress-related compounds including polyamines (spermine, spermidine, putrescine), ethylene, vitamin H (biotin), and phytosiderophores (polymers derived from non-protein amino acids deoxymugineic acid and mugineic acid involved in Fe uptake). Possible alterations in phytosiderophore biosynthesis are indicated by alterations in one enzyme of the Yang cycle, methylthioribose kinase like-1 (ssp 6513, cluster 2). Changes in methionine synthase and SAM synthase were reported in several proteomic studies dealing with abiotic stress responses (Yan et al., 2006; Vítámvás et al., 2012; Kosová et al., 2013). Alterations in methylthioribose kinase were already described by Patterson et al. (2007) in barley roots exposed to elevated boron, and by Fercha et al. (2013) in germinating wheat seedlings exposed to salinity. It is known that free metal ions can act as catalyzers of ROS formation in plant cells. Phytochelatins bind metal ions, thus preventing ROS formation.

### Stress- and defense-related proteins

A total of 14 proteins including proteins with chaperone and protective functions, as well as proteins directly involved in detoxification of ROS and xenobiotics, were identified in drought-treated barley crowns. Increased levels of the formation of xenobiotics is indirectly indicated by the enhanced accumulation of several glutathione-S-transferase (GST) isoforms (ssp 6108, cluster 1—glutathione-S-transferase I, subunit, ssp 7109, cluster 2—glutathione-S-transferase 6, chloroplastic, ssp 3116, cluster 1—glutathione-S-transferase F8, chloroplastic-like), which are known to conjugate xenobiotics with glutathione, resulting in the degradation of several xenobiotics. Increases in various GST classes has been reported by several proteomic studies dealing with stress (Kawamura and Uemura, 2003; Cui et al., 2005; Vítámvás et al., 2012; Budak et al., 2013; etc.). Moreover, several other roles for GST in protein regulation via S-glutathionylation as a post-translational modification have been reported in plants (Sappl et al., 2004; Dixon et al., 2010). Additionally, GST-catalyzed S-glutathionylation has also been reported for intermediates of several plant secondary metabolites such as tetrapyrroles, quercetin, glucosinolates, etc. (Dixon et al., 2010). Glutathione peroxidase (GPX; ssp 7006, cluster 4) catalyzes the reduction of peroxides and has cytoplasmic and membrane-associated forms. GPX belongs to the ROS scavenging enzymes; an increase in several ROS scavenging enzymes has been reported in most all the proteomic studies dealing with plant stress response, since imbalances in energy metabolism during stress treatments are associated with the enhanced risk of oxidative stress (Kosová et al., 2011; Vítámvás et al., 2012).

Protein spot 114 (cluster 3) was identified as heme-binding protein 2 (SOUL protein superfamily) involved in tetrapyrrole metabolism. In *Arabidopsis thaliana*, heme-binding protein TSPO is known to bind tetrapyrroles, and its dynamics of degradation seems to be affected by the level of delta-aminolevulinic acid and by abscisic acid. TSPO was found to attenuate plant cell porphyria by delta-aminolevulinic acid levels and the accumulation of tetrapyrroles (Vanhee et al., 2011). Therefore, our results also indicate the attenuation of porphyria in Amulet due to increase of heme-binding protein 2, delta-aminolevulinic acid dehydratase, and GSTs.

Cellular dehydration caused by decreased SWC induces the accumulation of several proteins with protective functions; these include hydrophilic LEA proteins (ssp 401, cluster 4—Late embryogenesis abundant protein Lea14-A), and proteins related to the heat shock protein (HSP) family (ssp 803, cluster 3—cytosolic HSP90) with a chaperone function. The increased accumulation of hydrophilic LEA proteins, chaperones, and HSP was reported in several proteomic studies (Caruso et al., 2008; Sarhadi et al., 2010; Kang et al., 2012; Budak et al., 2013; Kosová et al., 2013; Xu et al., 2013). However, some HSPs were also found to be decreased under abiotic stress treatment (e.g., HSP90 in cold-treated samples in Vítámvás et al., 2012). Wendelboe-Nelson and Morris (2012) and Ashoub et al. (2013) found a higher accumulation of HSP70 in tolerant genotypes of barley. Moreover, the opposite trends of obtained results compared to previous results on cold-acclimated cereals (Vítámvás et al., 2012; Hlaváčková et al., 2013; Kosová et al., 2013) in HSP90 (ssp 803; increase vs. decrease) and HSP70 (ssp 3804; decrease vs. increase), respectively, together with a decrease in the accumulation of cold shock protein (ssp 3006) could indicate a specific plant response to different abiotic stresses.

Protein spot 1119 (cluster 3) was identified as chitinase II, and revealed an increase under D1 with respect to the control. Chitinases belong to several classes of pathogenesis-related (PR) proteins including PR-3, 4, 8, and 11 classes (Edreva, 2005). Not only was an increase in chitinase accumulation found in cereals exposed to fungal pathogens (Yang et al., 2010; Eggert et al., 2011), but also exposed to several abiotic stresses such as cold (Sarhadi et al., 2010), salinity (Witzel et al., 2014), and others. Protein spot 8006 was identified as a PR17c precursor. An interaction with effector proteins secreted by fungal pathogens such as barley powdery mildew has been reported for PR17c in barley; however, the molecular function of PR17 proteins still remains to be well characterized (Zhang et al., 2012). Recently, PR17 was found to be increased in salt-treated barley (Witzel et al., 2014), which underlines our finding that this protein is also responsive to abiotic stress.

Protein spot 4303 (cluster 3) was identified as ricin B lectin 2, and it revealed an increase upon drought with respect to the control. Increased accumulation of ricin B lectin 2 was also found in the crowns of winter barley (Hlaváčková et al., 2013) and winter wheat (Kosová et al., 2013) exposed to cold.

Increased protein accumulation of r40c1 (ssp 8201, cluster 3) is supported by other results in drought-treated barley cultivars with contrasting drought tolerances. Protein r40c1 was found to have constant level in the drought-tolerant barley cv. Basrah, while being stress increased in the roots of the susceptible cv. GP (Wendelboe-Nelson and Morris, 2012). Therefore, the trend obtained in the accumulation of r40c1 supports the hypothesis that Amulet could be ranked as a genotype sensitive to drought. Moreover, an increased dephosphorylation was found in the putative r40c1 protein in drought-treated rice (Ke et al., 2009).

### Phytohormone metabolism

IAA-amino acid hydrolase ILR1-like protein 1 (ssp 3401, cluster 6) catalyzes a reversible IAA inactivation. Certain IAA-amino acid conjugates inhibit root elongation and therefore, the decrease of the protein revealed upon drought indicates an increase in root development (LeClere et al., 2002).

### Flavonoid metabolism

Flavonoids represent a group of secondary plant metabolites containing at least two phenolic rings in their molecules. Flavonoids display several antioxidant and antimicrobial functions; thus, playing an important role in the plant stress response. However, in our study, the enzymes of flavonoid metabolism showed a decrease after drought treatments but flavoprotein wrbA-like isoform 1 (ssp 7110, cluster 2). Flavonoid biosynthesis in plants is realized from malonyl-CoA via the phenylpropanoid pathway to yield tricetin. Tricetin is then sequentially O-methylated by tricin synthase (ssp 112, cluster 1), using SAM as a methyl donor to yield tricin. The decrease of isoflavone reductase (ssp 1213, cluster 6) under drought treatments is quite interesting since isoflavone reductase is a NADPH-dependent enzyme involved in the biosynthesis of defense-related isoflavonoid phytoalexins (Oommen et al., 1994). To our knowledge, no proteome study of drought-treated barley that revealed differential accumulation of flavonoid metabolism enzymes was published. However, some studies revealed differential changes in these proteins under salt treatment in plants. Flavone-O-methyltransferase was reported to be decreased upon salinity, with respect to the control, in germinating wheat seedlings by Fercha et al. (2014). An increase in isoflavone reductase was reported in salt-treated pea (Kav et al., 2004).

### Transport and cytoskeleton-related proteins

Annexin (ssp 8202, cluster 1) is a soluble protein that interacts with plasma membrane phospholipids. Monomeric annexins can form oligomeric channels enabling ion transport through plasma membrane, and they are also involved in vesicular trafficking and calcium signaling via MAPK cascade (for a review, see Laohavisit and Davies, 2011). An increase in annexin abundance was found also in salt-treated potato (Aghaei et al., 2008) and tomato (Manaa et al., 2011) plants, indicating their role in abiotic stress signaling.

Protein spot ssp 8708 (cluster 7) is identified as dynamin-related protein 5A-like protein, which belongs to the dynamin M family. Plant dynamins are GTPases, which are involved in clathrin-mediated endocytosis process, as well as in vesicle transport between TGN and the plasma membrane. They also form a ring in the plant plastid division process; thus, the results could indicate reduced plant cell division (Bednarek and Backues, 2010). However, up to now, no differential protein changes in cereal dynamin were published in drought treated plants.

Chloroplastic protein TOC75 (ssp 8801, cluster 6) is a part of the TOC transmembrane channel in the outer chloroplast membrane. TOC75 is directly involved in protein-protein interaction and transport (Andrès et al., 2010). In our study, the protein level of TOC75 was found to decrease upon drought with respect to the control, which corresponds to the decrease in OEE proteins as components of photosystem II when observed under stress.

### Quantitative changes between drought conditions

Not only were differences found in physiological parameters and in the density of protein spot accumulations between drought and control conditions, but also between both drought conditions. The drought treatments were clearly distinguished at the level of cellular dehydration (WSD and OP values); however, Δ^13^C and DHN5 relative accumulation did not reveal significant differences between the two treatments. Nonetheless, the analyses of each protein spot density should reveal detailed information about plant response to abiotic stress conditions. Based on revealed differential changes in identified proteins, it could be hypothesized that the two drought treatments differed in their intensity, which has been mirrored with some components of energy metabolism (glycolytic enzymes, ATP metabolism) and protein degradation (proteasome subunits). However, with regards to the D2 treatment, several protein spots have shown a relatively high variability in spot density between the four biological replicates, indicating that the D2 treatment may represent a threshold between plant stress acclimation and stress damage with regard to the intensity of stress (e.g., an increase in spot 119 identified as proteasome subunit beta; or a decrease in spot 205 identified as chloroplast OEE1 protein, under D2 with respect to D1 treatments). Thus, the different biological processes under severe stress conditions related to plant damage or exhaustion could influence the proteome profile of D2 compared to D1. Therefore, different trends in the accumulation of a few protein spots were obtained (e.g., 2,3-bisphosphoglycerate-independent phosphoglycerate mutase-like, ssp 4708). However, the fact that most of protein spots showed the same trends of protein accumulation compared to control conditions, with higher significant differences in D2 than in D1 compared to the C (71 and 50, respectively), indicated that for plant survival careful regulation of the same biological processes and pathways are needed in both stress conditions.

## Conclusions

Due to precise quantification of proteome changes by analysis of 2D-DIGE gels, we were able to determine 105 differently accumulated spots, and 76 of these were successfully identified. Until now, no other barley-drought proteome study had analyzed such a large number of protein spots. The study on drought response in the spring barley cv. Amulet has revealed that both drought treatments profoundly affected plant growth and development (changes in glycine-rich RNA-binding proteins, cell division cycle protein 48-like, gibberellin receptor GID1L2, translation initiation factor eIF5A) as well as plant energy metabolism. An enhanced need for available energy resources during the acclimation to stress conditions is indicated by profound changes in ATP metabolism as a resource of macroergic phosphate bonds. However, an enhanced risk of oxidative stress, as a consequence of imbalances in energy metabolism, leads to a downregulation of aerobic metabolism (photosynthesis, Krebs (TCA) cycle, mitochondrial electron transport chain) with respect to anaerobic metabolism (glycolysis, alcoholic fermentation). An increased risk of protein damage leads to an increase in several subunits of the proteasome complex and several protective proteins (cold shock protein, LEA-14A). Moreover, the metabolism of several stress-related metabolites (SAM metabolism—SAM as a precursor of polyamines, ethylene, phytosiderophores; flavonoid metabolism—flavonoids as protective pigments (anthocyanins) and cofactors of electron transport chain components) were significantly affected. In addition, the abundances of several proteins involved in cytoskeleton organization, protein and ion transport, etc., were affected by drought. Analysis of the obtained proteome changes demonstrated the possibility of the proteomics method used (2D-DIGE) for the evaluation of plant sensitivity or tolerance to abiotic stresses (i.e., for protein phenotyping of drought plant response). The enhanced severity of the D2 treatment was also observed at the proteome level as indicated by the differential abundance of several proteins involved in energy metabolism (glycolytic enzymes, ATP metabolism) and protein degradation (proteasome subunits); this was also validated on the physiological level (WSD and OP). Moreover, the high variability in the relative protein abundance (e.g., ssp 7109, GST6) between the four biological replicates in D2 treatment indicates an increased imbalance in cellular homeostasis in the D2 treatment, indicating a threshold between drought acclimation and damage under D2 conditions. Therefore, the wider comparison of protein abundances between other studies and ours (especially ssp 205, 401, 803, 2303, 3008, 4501, 7403, 8201, and 8201) focused on barley drought-induced proteome changes (Wendelboe-Nelson and Morris, 2012; Ashoub et al., 2013; Ghabooli et al., 2013; Kausar et al., 2013) can prove Amulet sensitivity to drought solely on the results of proteomic analysis.

For future protein phenotyping for drought plant response, the repeating and significant trends in protein spot accumulation under both drought conditions should be interesting to test. From the four protein spots that revealed a continuous significant increase under C, D1, and D2 treatments, only two spots (ssp 4614—UDP-glucose 6-dehydrogenase, cluster 3; and ssp 7006—glutathione peroxidase, cluster 3) were identified. However, all four (i.e., also ssp 7001 and 8104) could be good candidates for testing of their protein phenotyping capacity together with proteins that were significantly distinguished in both drought treatments.

### Conflict of interest statement

The authors declare that the research was conducted in the absence of any commercial or financial relationships that could be construed as a potential conflict of interest.
